# The effects of inhaled multi-walled carbon nanotubes on blood pressure and cardiac function

**DOI:** 10.1186/s11671-018-2603-5

**Published:** 2018-07-03

**Authors:** Wen Zheng, Walter McKinney, Michael L. Kashon, Daniel Pan, Vincent Castranova, Hong Kan

**Affiliations:** 1 Health Effects Laboratory Division, National Institute for Occupational Safety and Health, Morgantown, WV 26505 USA; 2 Department of Pharmaceutical Sciences, School of Pharmacy, West Virginia University, Morgantown, WV 26506 USA; 3 Health Effects Laboratory Division, Pathology and Physiology Research Branch, National Institute for Occupational Safety and Health, 1095 Willowdale Road, Morgantown, WV 26505 USA

**Keywords:** Inhalation, Nanoparticles, Multi-walled carbon nanotubes, Blood pressure, Cardiac function

## Abstract

**Background:**

Heart rate variability (HRV) as a marker reflects the activity of the autonomic nervous system. The prognostic significance of HRV for cardiovascular disease has been reported in clinical and epidemiological studies. Our laboratory has reported alterations in rat heart rate variability (HRV) due to increasing activity of both sympathetic and parasympathetic nervous system after pulmonary exposure to multi-walled carbon nanotubes (MWCNTs). This suggests that pulmonary inhalation of engineered nanoparticles (ENs) may lead to functional changes in the cardiovascular system. The present study further investigated the effects of inhaled MWCNTs on the cardiovascular system and evaluated the correlation between the alterations in HRV and changes in cardiovascular function.

**Methods:**

Male Sprague-Dawley rats were pre-implanted with a telemetry device and exposed by inhalation to MWCNTs for 5 h at a concentration of 5 mg/m^3^. The electrocardiogram (EKG) and blood pressure were recorded in real time by the telemetry system at pre-exposure, during exposure, and 1 and 7 days post-exposure. In vivo cardiac functional performance in response to dobutamine was determined by a computerized pressure-volume loop system.

**Results:**

Inhalation of MWCNTs significantly increased both systolic and diastolic blood pressure and decreased heart rate in awake freely moving rat. Additionally, inhalation of MWCNTs also reduced cardiac stroke work, stroke volume, and output in response to dobutamine in anesthetized rats.

**Conclusions:**

Inhalation of MWCNTs altered cardiovascular performance, which was associated with MWCNT exposure-induced alterations in the sympathetic and parasympathetic nervous system. These findings suggest the need to further investigate the cardiovascular effects of inhaled MWCNTs.

## Background

With potential wide industrial application and increasing production, the chance of exposure to nanomaterials has increased in many industry sectors. Therefore, the adverse health effects of exposure to nanomaterials have received great attention. Animal studies indicated that short-term pulmonary exposure to engineered nanoparticles can cause a severe or minor inflammatory reaction in the lung depending on the physical and chemical properties of nanomaterial tested. In addition, pulmonary exposure to carbon nanotubes (CNTs) has been linked to lung fibrosis and cancer promotion [1–5]. Although the recent emphasis on occupational exposure to nanomaterials has focused more on lung disease and carcinogenesis and less on the cardiovascular system, evidence from epidemiological and recent animal studies strongly indicate that pulmonary exposure to nanomaterials can affect the cardiovascular system by nanoparticle-induced inflammation, translocation, and/or neuron regulation [6–9]. Our studies have found that nanoparticles from ultrafine titanium dioxide (UFTiO_2_) and multi-walled carbon nanotubes (MWCNTs), at doses that cause a minor acute inflammatory reaction in the lung, can transiently increase neurotransmitter synthesis in the peripheral neurons [8] and lead to the alterations in the activity of the autonomic nervous system (ANS) [10]. In addition, we also reported previously that direct exposure of isolated cardiomyocytes to UFTiO_2_ did not alter biological activity of cardiomyocytes [11]. Taken together, our studies strongly suggested that some nanomaterials, at doses which exhibit a minor acute effect in the lung, affect the cardiovascular system by influencing the neuronal system rather than by the direct translocation of nanoparticles to the heart.

The ANS plays a critical role in maintaining normal cardiovascular function. ANS disturbance can result in functional disorders in the cardiovascular system, which may result in hypertension, stroke, or cardiac arrhythmia [12–15]. Epidemiological studies have supported the potential for inhaled nanoparticles to induce cardiovascular sequela. For example, particulate air pollution inhalation for just a few hours has increased cardiovascular disease-related mortality and morbidity by altering the ANS balance in people, particularly those with pre-existing cardiovascular conditions [16]. Epidemiological studies also indicate that ultrafine particles (UFP) may contribute significantly to the cardiovascular effects of particulate air pollution, partly because of the relatively more efficient alveolar deposition of UFP vs fine particles [17]. Larger airborne particles that deposit in the conducting airway can be removed rapidly by the mucociliary escalator, a major mechanism for pulmonary clearance. However, nano-sized particles may bypass this first defense system, penetrate deeply in the trachea and the lung to stimulate sensory neuronal endings persistently. We demonstrated previously that inhaled MWCNTs significantly altered heart rate variability (HRV) [10]. The present study used a rat model to further elucidate the influence of pulmonary exposure to MWCNTs on the function of the cardiovascular system and correlate these alterations to the activity of the ANS.

## Methods

### Animal

Male Sprague-Dawley (Hla: (SD) CVF) rats from Hilltop Lab Animals (Scottdale, PA, USA), weighing 275–300 g and free of viral pathogens, parasites, and mycoplasmas, Helicobacter and cilia-associated respiratory (CAR) bacillus were used for all experiments. The rats were acclimated for 1 week after arrival and housed in filter top cages under controlled temperature and humidity conditions and a 12-h light/12-h dark cycle. Food (Teklad 7913) and tap water were provided ad libitum. The animal facilities are specific pathogen-free, environmentally controlled, and accredited by the Association for Assessment and Accreditation of Laboratory Animal Care International (AAALAC). All animal procedures used during the study have been reviewed and approved by the National Institute for Occupational Safety and Health Animal Care and Use Committee.

#### Pulmonary MWCNT Inhalation Exposure

MWCNTs were obtained from Hodogaya Chemical Company (MWCNT-7, lot no. 061220-31). Male Sprague-Dawley rats (250–300 g) were exposed to a MWCNT aerosol (5 mg/m^3^) for 5 h. Rats were placed individually in sealed cages which were connected to the main exposure chamber (used as a mixing chamber for this study) through anti-static flexible tubing. Gilian gilair-5 R basic air sampling pumps (Sensidyne, St. Petersburg, FL 33716 USA) were attached to the sealed cages to pull either aerosol of MWCNTs from the main exposure/mixing chamber or filtered air (control group) into the sealed cage at a flow rate of 1.25 l/min. Particle mass size distribution of MWCNT aerosol in the sealed cage was determined by a cascade impactor (MOUDI, Models 110 and 115, MSP Co., Shoreview, MN). The MWCNT mass concentration was determined by a physical gravimetric analysis with Teflon filters. The aerosol generation system, exposure chamber, and physical characterization of the MWCNT aerosol have been described elsewhere [10, 18, 19]. Using a deposition fraction of 1.5 or 2.7% and an average minute ventilation of 186 ml/min [5], the total lung burden with our exposure scheme is calculated as 5 mg/m^3^ (exposure concentration) ×  186 ml/min (minute ventilation) ×  10^− 6^ m^3^/ml (volume conversion) ×  300 min (exposure duration) × 1.5 or 2.7% (alveolar deposition fraction), which is approximately equal to 4.2 or 7.5 μg MWCNTs in rats. It only takes 14–25 days of exposure to reach the same lung burden if a worker exposed to MWCNTs at a level of 40 μg/m^3^, which is a feasible human occupational exposures [19, 20].

#### Telemetry Transmitter Implantation

Before the surgery, rats were kept separately, quiet, and handled gently to avoid distress. Surgical instruments and supplies were autoclaved, and aseptic technique was used throughout the surgical procedure. Anesthesia was induced with 3% isoflurane and 1 l per minute of oxygen in an induction chamber and maintained at 2% isoflurane and ½ liter per minute of oxygen during the surgery. A temperature-controlled heating pad was used to maintain normal body temperature of the rats which was monitored via an anal probe during the entire procedure. Cardiopulmonary responses were examined as an intraoperative monitoring technique along with the spinal reflexes to determine the proper depth of anesthesia. The incision sites were clipped and then aseptically prepared with povidone-iodine, followed by 70% alcohol. A midline abdominal incision was made, and the abdominal aorta was exposed by using sterile cotton swabs. The pressure catheter of the telemetry transmitter (HD-S21, Data Sciences International, St. Paul, MN) was inserted into the abdominal aorta and guided upstream. Tissue adhesive (Vetbond, 3M Animal Care Products, St Paul, MN) was used to secure the catheter and obtain hemostasis. The body of the telemetric device was positioned underneath the abdominal wall on the left lateral side of the incision and was secured in place by suturing to the abdominal muscle using 4–0 non-absorbable suture (Surgical Specialties Corporation, Wyomissing, PA). Post-operative care included 5 mg/kg of meloxicam (Metacam, Boehringer Ingelheim Vetmedica, Inc. St. Joseph, MO) administered subcutaneously for pain relief, once a day for 4 days. The general condition, body weight, and food and water consumption of the rats were closely monitored. Rats had a period of 3 weeks convalescence before data acquisition and inhalation exposure.

#### In Vivo Hemodynamic Measurements

Left ventricular function in response to dobutamine after exposure to MWCNTs was evaluated by a pressure-volume loop catheter placed in the left ventricle in the anesthetized rat. At 1 and 7 days post-exposure, the rat was anesthetized with 3% isoflurane with 2 l per minute of oxygen in an induction chamber and maintained at 1–2% isoflurane with 1 l per minute of oxygen during the surgery. Cardiopulmonary response (heart rate, breath rate, and depth) and toe pinched spinal reflex were examined as intraoperative monitoring techniques. The normal body temperature was maintained by a temperature-controlled heating pad and was monitored via an anal probe during the entire procedure. The rat was placed in dorsal recumbent position, and the incision sites were clipped and then aseptically prepared with povidone-iodine, followed by 70% alcohol. Millar’s Mikro-Tip® ultra-miniature PV loop catheter (SPR-901, Millar, Inc. Houston, TX) was inserted into the left ventricle through the carotid artery. The correct position of the catheter tip in the left ventricle was confirmed by the waveform of pressure-volume loop visualized on a computer monitor. After stabilization for 20 min, the signals of left ventricular function were continuously recorded at a sampling rate of 1000 samples/s using a PV conductance system (MPVS-Ultra, Millar Instruments, Houston, TX, USA) connected to the PowerLab 4/30 data acquisition system (AD Instruments, Colorado Springs, CO, USA). Dobutamine, USP grade (Hospira, Inc., Lake Forest, IL), was prepared in pharmaceutical sterile saline solution (1.25, 2.5, 5, 10 μg/kg/50 μl) and applied through jugular vein by a Pump 11 Elite Programmable Syringe Pump (Harvard Apparatus, Holliston, MA, USA) for 30 s for each dose.

#### Data Acquisition and Analysis

Blood pressure of awake freely moving rats was recorded continuously for 24 h before exposure, during the MWCNT exposure, 1 and 7 days post-exposure. On the exposure day, rats were allowed to acclimate to the chamber for 30 min, then 5-h (9 am–2 pm) continuous recordings were made during exposure. Blood pressure data from each animal were collected and then exported (Dataquest ART analysis software; Data Sciences International) to an Excel spreadsheet program (Excel 2010, Microsoft Corporation, Seattle, WA). Systolic blood pressure (SBP), diastolic blood pressure (DBP), and mean blood pressure were averaged over the course of 5 h exposure (9 am–2 pm) for comparisons between control and MWCNT exposure groups.

#### Statistical Analysis

Data were compared using two-way (treatment by day) repeated measures analysis of variance. Subsequent pairwise comparisons were tested using Fishers LSD. All data were analyzed using SAS software (Version 9.3), and differences were considered statistically significant at the level of *p* < 0.05. The values in the figures were expressed as the mean ± SE.

## Results

**Fig. 1:**
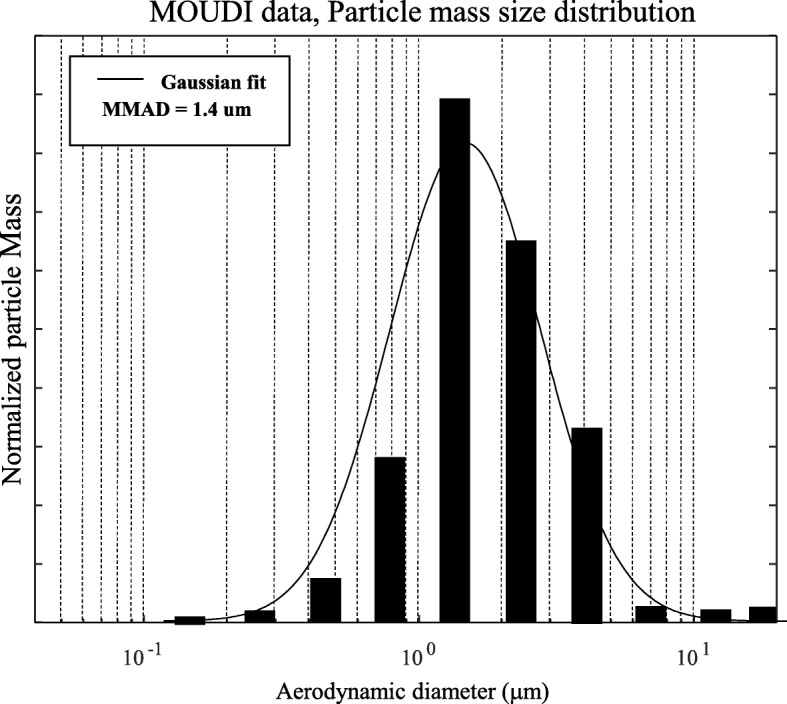
A typical size distribution of the MWCNT aerosol in the sealed exposure cage which indicates a mass median aerodynamic diameter of 1.4 μm

In this study, the particle mass size distribution and mass concentration of MWCNT aerosol in the sealed exposure cage were determined. The results indicate a mass median aerodynamic diameter of 1.4 μm (Fig. 1) and the MWCNT mass concentration of 5 mg/m^3^ (data not shown).

**Fig. 2:**
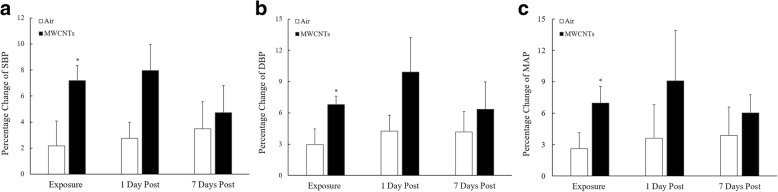
**a** Bar graph depicting a percentage change of systolic blood pressure (SBP) during the exposure period and at 1 and 7 days after exposure from the basal level before exposure (pre-exposure control vs MWCNTs: 127.0 ± 3.0 vs 127.6 ± 1.7 mmHg). **b** Bar graph depicting a percentage change of diastolic blood pressure (DBP) during the exposure period and at 1 and 7 days after exposure from the basal level before exposure (pre-exposure control vs MWCNTs: 85.1 ± 2.0 vs 86.9 ± 1.2 mmHg). **c** Bar graph depicting a percentage change of mean blood pressure (MAP) during the exposure period and at 1 and 7 days after exposure from the basal level before exposure (pre-exposure control vs MWCNTs: 99.1 ± 2.3 vs 100.4 ± 1.4 mmHg). Each value represents the mean ± SE of eight rats. *P* < 0.01 compared with control group (*)

The blood pressure was measured in freely moving rats instrumented with the telemetry and compared as percentage change from pre-exposure. Our results indicated that systolic, diastolic, and mean blood pressure were all significantly increased during the 5-h exposure period in the MWCNT-exposed group when compared with control group (Fig. 2a–c). At 1 day post-exposure, although the percent change in systolic, diastolic, and mean blood pressure in MWCNT-exposed group still remained higher than in the control group, the difference was not significant (Fig. 2a–c). At 7 days post-exposure, there was no difference in blood pressure observed between the two groups (Fig. 2a–c).

**Fig. 3:**
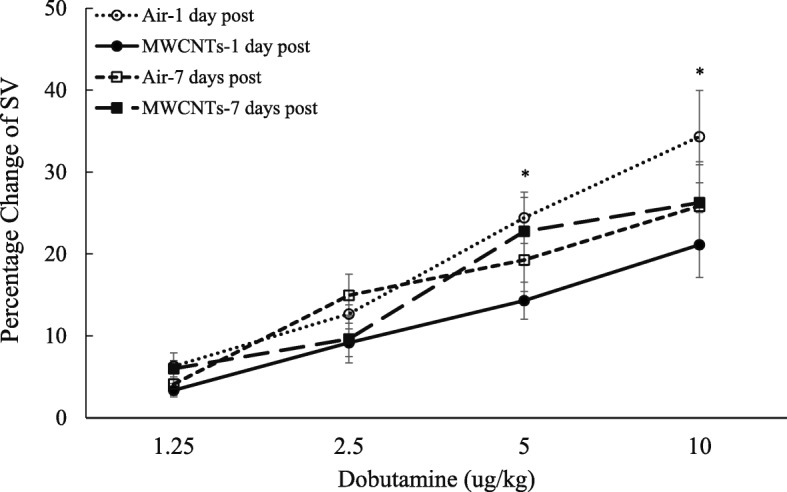
Line graph depicting a percentage change of stroke volume (SV) from the basal level before exposure (pre-exposure control vs MWCNTs at 1 day post: 109.3 ± 7.0 vs 106.7 ± 10.4 μl, control vs MWCNTs at 7 days post: 118.8 ± 5.7 vs 127.5 ± 3.7 μl). Each value represents the mean ± SE of eight rats. *P* < 0.01 exposed compared with control group at 1 day post-exposure (*)

**Fig. 4:**
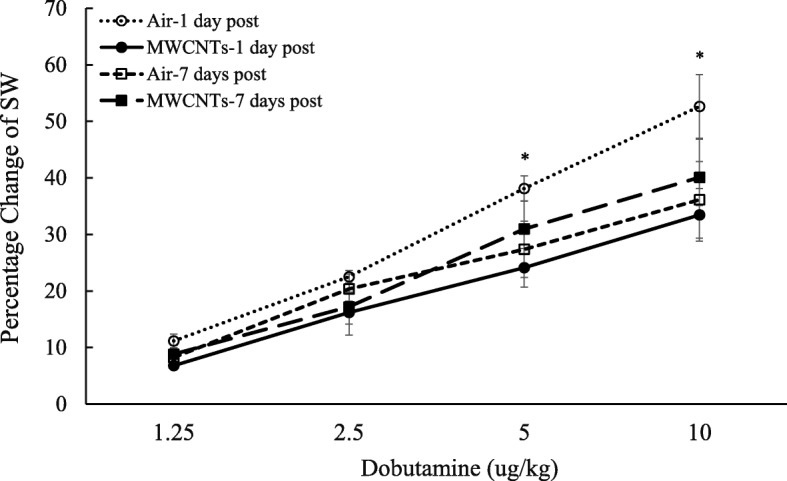
Line graph depicting a percentage change of stroke work (SW) from the basal level before exposure (pre-exposure control vs MWCNTs at 1 day post: 11276 ± 1165 vs 11,151.7 ± 727.9 mmHg × μl, control vs MWCNTs at 7 days post: 13245 ± 893.4 vs 13,644.2 ± 536.5 mmHg × μl). Each value represents the mean ± SE of eight rats. *P* < 0.01 exposed compared with control group at 1 day post-exposure (*)

**Fig. 5:**
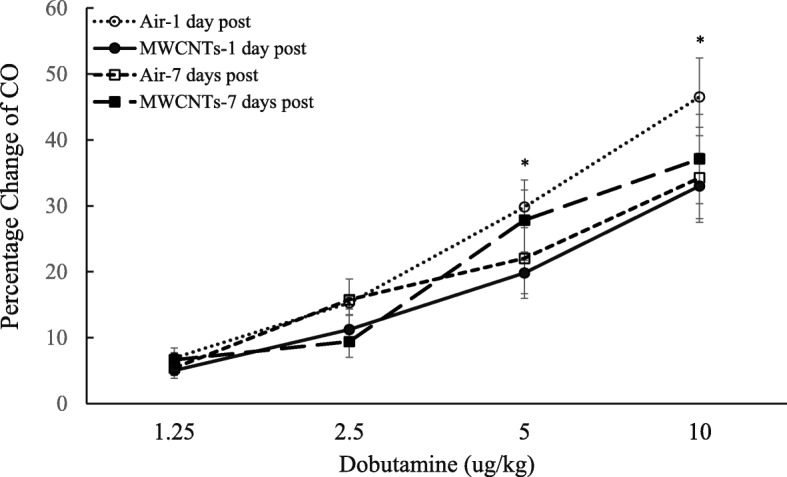
Line graph depicting a percentage change of cardiac output (CO) from the basal level before exposure (pre-exposure control vs MWCNTs at 1 day post: 42243.3 ± 4500.1 vs 40,556.6 ± 2308.8 μl/min, control vs MWCNTs at 7 days post: 44903.3 ± 2906.0 vs 46,210 ± 1624.8 μl/min). Each value represents the mean ± SE of eight rats. *P* < 0.01 exposed compared with control group at 1 day post-exposure (*)

**Fig. 6:**
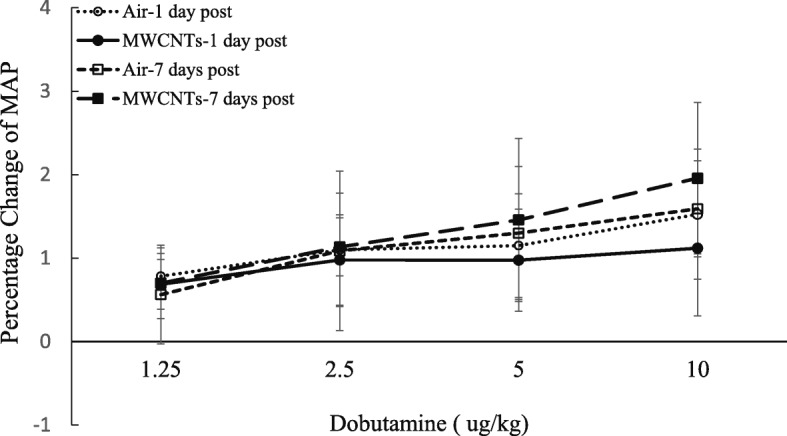
Line graph depicting a percentage change of mean blood pressure (MBP) from the basal level before exposure (control vs MWCNTs at 1 day post: 97.7 ± 2.8 vs 98.1 ± 2.6, control vs MWCNTs at 7 days post: 102.5 ± 4.2 vs 100.9 ± 5.5). Each value represents the mean ± SE of eight rats

Cardiac function after exposure to MWCNTs was evaluated by measuring left ventricular performance in response to increased doses of dobutamine in anesthetized rats at 1 and 7 days post-exposure. The results indicated that exposure to MWCNTs slightly depressed basal cardiac stroke volume(SV), cardiac stroke work (SW), and cardiac output (CO), but significantly reduced the responsiveness of stroke volume, stroke work, and cardiac output to increased dose of dobutamine at 1 day post-exposure (Figs. 3, 4, and 5). There was no difference observed between the two groups at 7 days post-exposure (Figs. 3, 4, and 5). The blood pressure in the presence of increased doses of dobutamine was also measured, and there was no difference between control and MWCNT-exposed groups (Fig. 6).

## Discussion

The role of autonomic nervous system (ANS) in regulation of cardiovascular function has been well studied [21]. We reported previously that pulmonary inhalation of MWCNTs alters heart rate variability (HRV) and reduced heart rate (HR) via increasing the activity of both the sympathetic and parasympathetic nervous system in the rats [10]. In workers, pulmonary exposure to TiO_2_ particles < 300 nm in diameter was associated with altered HRV, a change consistent with particle effects on the autonomic nervous system [22]. Accordingly, an epidemiological study confirmed that the ultrafine component of particulate matter in ambient air plays a key role in regulation of cardiovascular autonomic nervous activity [23]. However, the mechanisms by which the alterations in autonomic nervous system resulting from pulmonary exposure to ENs affect the cardiovascular function remain unclear. The sympathetic and parasympathetic nervous system tends to act in a reciprocal manner to regulate cardiovascular function. However, our findings indicate that the activity of both sympathetic and parasympathetic nerves was increased simultaneously following exposure to MWCNTs [10]. In order to elucidate the consequence of altered ANS activity following exposure to ENs in cardiovascular performance, blood pressure was recorded and analyzed from the same awake freely moving rats used for studying HRV as we reported previously [10]. Our results indicated that systolic, diastolic, and mean blood pressure all were elevated significantly during MWCNT exposure when compared with the control group (Fig. 2a–c) and remained somewhat higher (although not significantly) at 1 day post-exposure. The significantly higher blood pressure following pulmonary exposure to MWCNTs was not likely due to a stress reaction since the response was maintained throughout the 5-h exposure and differed from that of filtered air controls. A stress reaction usually results in a fight-or-flight response, a physiological reaction with increased blood pressure and accelerated heart rate and stronger cardiac contraction due to inhibitory effect on parasympathetic neuronal system. In our previous study, the activity of sympathetic nervous system was directly stimulated by MWCNT inhalation suggesting that a MWCNT-stimulated increase in the activity of sympathetic nervous system was responsible for the higher blood pressure following exposure to MWCNTs in the present study.

In the present study, our results suggest that the increased blood pressure during the exposure to MWCNTs was associated with reduced heart rate in awake freely moving rats compared with the control group (bordering on statistical significance (*p* = 0.054)) (data not shown)). The reduced heart rate observed during the exposure was consistent with our previous report that there was an increased parasympathetic nerve activity during the exposure to MWCNTs [10]. The evidence of a correlation between increased parasympathetic nervous activity and the effect on the cardiac performance following exposure to MWCNTs was supported by studying the basal cardiac performance and the cardiac responsiveness to dobutamine, a β-adrenergic receptor agonist, in anesthetized rats. At 1 day post-exposure, the basal cardiac activities of heart rate, cardiac output, and left ventricular end-systolic pressure were all lower in MWCNT-exposed rats, although the differences did not reach statistical difference (data not shown). The influence of increased parasympathetic nervous activity on the heart was further indicated as reduced responsiveness of stroke volume, cardiac work, and cardiac output to dobutamine (Figs. 3, 4, and 5). Dobutamine is a β-receptor agonist. Activation of β-receptors in the heart mimics a sympathetic effect. Therefore, the reduced responsiveness of cardiac performance to dobutamine could be due to a decrease of sympathetic nervous activity. However, in the present study, the reduced cardiac performance in the presence of dobutamine more likely resulted from an increased parasympathetic activity during MWCNT exposure since our previous study indicated that the sympathetic nervous activity remained high following MWCNT exposure in awake freely moving rats [10]. Although there was a timing difference for measurement of blood pressure and cardiac function from conscious, freely moving rats and anesthetized rats, reduced heart rate in conscious rats together with decreased responsiveness of cardiac function to dobutamine (Figs. 3, 4, and 5) implies an elevated parasympathetic activity and its effects on the heart after exposure to MWCNTs.

There are two mechanisms that may contribute to decreased heart rate and cardiac performance along with an increased blood pressure that occurred in this study. One is a baroreceptor reflex response which is well established. The second is a direct increase of the parasympathetic neuronal output in the cardiovascular center after inhalation of MWCNTs as we reported previously [10]. Both mechanisms involve the parasympathetic nervous system but with different pathways. It is well known that increased blood pressure can excite the baroreceptor by increasing its basal rate of action potential generation and send the signal to the nucleus of the tractus solitarius (NTS), which in turn inhibits the vasomotor center and stimulates the vagal nuclei [24, 25]. The end-result is to reduce the heart rate and cardiac contractility, which keeps the blood pressure in a narrow fluctuation range. In our study, reduced heart rate and cardiac performance were associated with significantly higher blood pressure following MWCNT exposure in conscious rats, which could be due to high blood pressure triggering the baroreceptor reflex. However, in anesthetized rats, we found no difference in the basal blood pressure between the control and exposure groups (control vs MWCNTs: mean blood pressure 98.6 vs 97.9 mmHg), most likely due to the impact of anesthesia [26]. The cardiac performance in MWCNT exposure group was relatively weak at the basal level comparing with control group (see the legends of Figs. 3, 4, and 5). Interestingly, the responsiveness of stroke volume, cardiac work, and cardiac output to increased dose of dobutamine was significantly weaker in rats exposed to MWCNTs, while there was no difference in the blood pressure measured simultaneously with the cardiac function in response to dobutamine between the control and MWCNT groups (Fig. 6). These observations excluded the role of baroreceptor reflex and strongly suggested that pulmonary exposure to MWCNTs can increase the activity of parasympathetic nervous system through a mechanism other than the baroreceptor reflex. The evidence of direct stimulation of parasympathetic nervous activity by carbon nanotubes was also observed in another animal study [27]. This study found that intratracheally instilled single-walled carbon nanotubes reduced heart rate without an increase in blood pressure in rats [27]. Considering the rapid onset during the exposure and the transient effects on the ANS, blood pressure, and cardiac performance, our study also excludes the possible role of nanoparticle-induced inflammation and nanoparticle translocation in regulation of cardiovascular function and supports the hypothesis that pulmonary exposure to nanoparticles may directly affect cerebral areas that are responsible for autonomic control, which in turn affects cardiovascular function.

Our study indicates that the effect of MWCNT-induced alterations in ANS on the cardiovascular system is apparently based on the distribution of autonomic nerves. In the vasculature system, the blood vessels mainly are innervated by sympathetic nerves, the majority of these sympathetic nerves release norepinephrine (NE) that binds to α_1_-adrenergical receptors to cause vessel constriction. In the body, there are only few types of blood vessels that are innervated by parasympathetic cholinergic or sympathetic cholinergic nerves, both of which release acetylcholine (ACh) that binds to muscarinic receptors to cause vessel dilation. Therefore, the overall effect of an increase in both sympathetic and parasympathetic nervous activity is to increase blood pressure by vessel constriction. The heart is innervated by both parasympathetic and sympathetic fibers which work in a reciprocal fashion to modulate heart rate (chronotropy), force of contraction (inotropy), and relaxation (lusitropy) [28, 29]. Exposure to MWCNTs induced slower heart rate and reduced stroke volume, stroke work, and cardiac output in response to dobutamine (Figs. 3, 4, and 5), suggesting that increased activity of parasympathetic nervous system was dominant in controlling heart rate and cardiac performance following pulmonary exposure to MWCNTs, even in the face of increased sympathetic activity.

The present study was the first to report that exposure to MWCNTs induced alterations in ANS, which can significantly affect cardiovascular function. Although the effects of inhaled MWCNTs on the blood pressure, heart rate, and cardiac function were observed predominantly during the exposure period, and the transient increase in blood pressure and a depressed cardiac performance adapt quickly in healthy animals, such transient changes in the cardiovascular function could be a risk factor in triggering a cardiovascular event in those with pre-existing cardiovascular conditions such as heart failure and hypertension. A recent in vivo ischemia/reperfusion (I/R) study indicated that pulmonary exposure to MWCNTs significantly increases I/R injury even in the absence of a significant circulatory inflammation response [30]. It has been well studied that a disturbance in autonomic nervous system can increase I/R injury that results in more cardiac tissue damage during cardiac ischemia [31]. During heart failure, the heart does not pump sufficient blood to the lung for oxygen exchange and to the rest of body to maintain proper organ function due to weakness of the cardiac muscle. In that compromised state, further increasing blood pressure and reducing cardiac contractility by MWCNTs exposure could result in worsening an already disrupted cardiovascular function and organ perfusion. Our study clearly indicates that MWCNT exposure can stimulate ANS activity that is associated with an alteration in cardiovascular function. The observation from our study may be relevant to the conclusion from the American Heart Association that exposure to particulate matter < 2.5 μm in ambient air for only a few hours or weeks can trigger cardiovascular disease-related mortality and morbidity in people with pre-existing cardiovascular conditions [16].

## Conclusions

The observations in the present study provide fundamental evidence to support our previous findings and the hypothesis that pulmonary exposure to nanoparticles can affect cardiovascular function due to the alterations in ANS activity. In conclusion, our study indicates that exposure to MWCNT-induced alterations in the ANS can significantly affect cardiovascular function. Further studies are warranted to investigate whether the transient alterations in cardiovascular function can cause more severe adverse impact on those with pre-existing cardiovascular conditions.

## Abbreviations

CNTs: Carbon nanotubes
CO: Cardiac output
DBP: Diastolic blood pressure
EKG: Electrocardiogram
ENs: Engineered nanoparticles
HR: Heart rate
HRV: Heart rate variability
MAP: Mean blood pressure
MWCNTs: Multi-walled carbon nanotubes
SBP: Systolic blood pressure
SV: Stroke volume
SW: Stroke work
